# Prediction of protein solubility based on sequence physicochemical patterns and distributed representation information with DeepSoluE

**DOI:** 10.1186/s12915-023-01510-8

**Published:** 2023-01-24

**Authors:** Chao Wang, Quan Zou

**Affiliations:** 1 School of Software Engineering, Chengdu University of Information Technology, Chengdu, China; 2 Institute of Fundamental and Frontier Sciences, University of Electronic Science and Technology of China, Chengdu, China

**Keywords:** Protein solubility, Feature embedding, Machine learning, Interpretation

## Abstract

**Background:**

Protein solubility is a precondition for efficient heterologous protein expression at the basis of most industrial applications and for functional interpretation in basic research. However, recurrent formation of inclusion bodies is still an inevitable roadblock in protein science and industry, where only nearly a quarter of proteins can be successfully expressed in soluble form. Despite numerous solubility prediction models having been developed over time, their performance remains unsatisfactory in the context of the current strong increase in available protein sequences. Hence, it is imperative to develop novel and highly accurate predictors that enable the prioritization of highly soluble proteins to reduce the cost of actual experimental work.

**Results:**

In this study, we developed a novel tool, DeepSoluE, which predicts protein solubility using a long-short-term memory (LSTM) network with hybrid features composed of physicochemical patterns and distributed representation of amino acids. Comparison results showed that the proposed model achieved more accurate and balanced performance than existing tools. Furthermore, we explored specific features that have a dominant impact on the model performance as well as their interaction effects.

**Conclusions:**

DeepSoluE is suitable for the prediction of protein solubility in *E. coli*; it serves as a bioinformatics tool for prescreening of potentially soluble targets to reduce the cost of wet-experimental studies. The publicly available webserver is freely accessible at http://lab.malab.cn/~wangchao/softs/DeepSoluE/.

**Supplementary Information:**

The online version contains supplementary material available at 10.1186/s12915-023-01510-8.

## Background

Protein solubility is a critical prerequisite for successful heterologous protein expression in host cells, such as *Escherichia coli* (*E. coli*) [1]. Solubility deficits result in protein aggregates, which affect protein biological activity, cause recombinant protein pipelines to fail, hamper protein-based drug development [2, 3], and cause more than forty diseases [4]. Unfortunately, in most cases, heterologous expression fails due to the formation of inclusion bodies, as solubility depends not only on protein physicochemical properties [5] but also on host type and the strict internal cellular environment, such as pH, ionic strength, and temperature [6, 7]. Furthermore, growth media, gene expression level [1], molecular chaperones, and solubility-enhancing tags also have a strong influence on protein solubility [8]. To date, solubility is still an inevitable barrier in protein science and industry, where only nearly a quarter of proteins can be successfully expressed in soluble form (http://targetdb.rcsb.org/metrics/).

In view of the low success rate for heterologous protein expression and the explosive growth of protein sequences, prescreening of potentially soluble targets is urgently needed before wet experiments. Over the past decades, several solubility prediction models have been developed based on intrinsic protein properties. Additional file 1: Table S1 summarizes the existing tools for solubility prediction and covers a wide range of aspects, including training and evaluation datasets, feature descriptors and classifiers, evaluation methods, and tool availability. According to the operating algorithm, these methods were roughly grouped into three categories: (i) statistical-based models (e.g., statistical correlation and arithmetic mean), such as the revised Wilknson-Harrison model (rWH) [1, 9], ccSOL omics [10], and SWI [5]; (ii) conventional machine learning (e.g., support vector machines and naïve Bayes)-based models, such as PROSO [8] and SoluPort [11]; and (iii) neural network-based models, such as DeepSol [12] and SKADE [13].

Although these in silico bioinformatics models have greatly contributed to protein science studies, the performances achieved by existing predictors are still far from satisfactory. The purpose of this study is to address this problem. We developed a novel tool, DeepSoluE, for protein solubility prediction. The physicochemical features and distributed amino acid representative information were combined to uncover sequence patterns in multiple aspects, and a genetic algorithm was used for optimal feature subset selection. Then, LSTM networks were applied to integrate feature information and to perform classification. We demonstrate that the proposed predictor DeepSoluE outperforms the existing methods in protein solubility prediction.

## Results and discussion

### Descriptor parameter optimization and feature selection

The feature vector dimensions of two of the five physicochemical descriptors, i.e., QSorder and APAAC, are dependent on the algorithm parameters. To make each type of feature as informative as possible, the related parameters were optimized before they were used for feature optimization. The parameter search range and the optimal value are listed in Additional file 1: Table S2. After the parameter value is determined, the combined feature dimension generated by the five physicochemical-based descriptors is 523D. To reduce the computing complexity and avoid the overfitting issue of the machine learning model, the genetic algorithm was applied to choose the optimal feature subset from the combined features. The number of populations was set to 200, and the chromosome length and the number of generations were set to 100 and 500, respectively (refer to the “Methods” section for details). To evaluate the effectiveness of the genetic algorithm for informative feature identification, four other widely used two-step feature selection strategies were used for comparison. In the first step, four types of feature importance values, calculated by random forest (RF), light gradient boosting machine (LGB) [14], F-score, and MRMD [15], were calculated to yield four descending order lists. In the second step, for each feature list, the optimal feature subsets were selected using the sequential forward search (SFS) method [16]. Finally, the feature subset leading to the model with the highest AUC value is retained as the optimal feature subset.

**Fig. 1:**
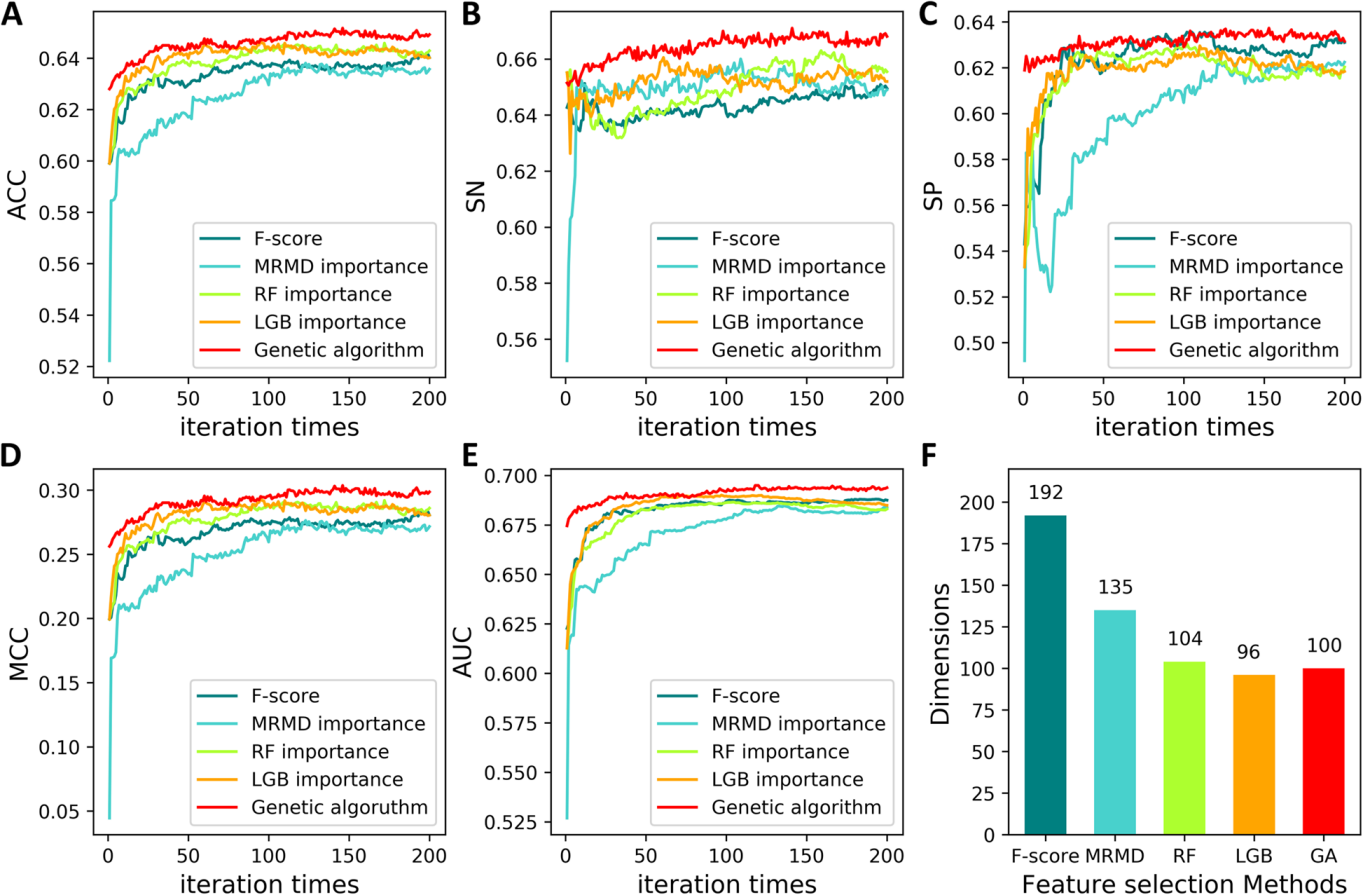
Comparison of different feature selection methods. **A**–**E** Metrices value and feature dimensions based on five feature selection strategies. **F** Feature dimensions of optimal feature subsets based on the metric AUC of the five feature optimization methods. GA: genetic algorithm.

The results of the above five feature selection strategies are presented in Fig. 1. Of note, the feature dimension of the genetic algorithm is fixed to 100D as the demand of the algorithm structure, while the dimensions of the remaining four feature importance-based SVM models linearly increase with the number of iterations from 1 to 200. Generally, the five metric values are gradually increased, and the maximum scores are obtained at approximately 100 iterations. Specifically, among the five feature selection strategies, the genetic algorithm resulted in the best performance when evaluated by ACC, SN, SP, MCC, and AUC. LGB (importance_type=’gain’) and RF importance-based methods are ranked at the second level, and MRMD- and *F*-score-based feature selection methods are proven to be the least effective strategies. It can be observed from Fis. 1A–E that the changing trend of the five metrics is not completely synchronized, so the AUC value is used to choose the best feature subset. As shown in Fig. 1E, the maximum AUC reached 0.6949 at the 117th iteration; therefore, the genes, namely, the features, retained in the 117th generation were kept as the optimal feature subset of the five physicochemical descriptors. The feature dimensions corresponding to the maximum AUC of the above five feature optimization methods are shown in Fig. 1F, where the LGB (96D) and genetic algorithm (100D) methods exhibited the lowest dimensions. Considering the model performance and feature dimension, it can be concluded that the genetic algorithm-based feature selection strategy outperformed the other four methods.

### Distributed representation of amino acid fragments

All training protein sequences were divided into a k-mer corpus, and each k-mer was embedded into a 100-dimensional feature using word2vec with a skip-gram model. In this process, two critical parameters, namely, the sliding window (length *k* of the *k*-mer) and the number of surrounding words (window size w from word2vec), were optimized. The *k* value was varied from 2 to 6, and the *w* value was varied from 1 to 7. The ACC values for all possible combinations of k and w are depicted in Additional file 2: Fig. S1. We observed that the ACC values gradually decreased with the value of k, while the w value had less impact on the ACC value. Taken together, the parameters resulting in the maximum ACC value were adopted for the final skip-gram model, which is based on a k-mer length of 3 and a window size of 2.

### DeepSoluE model

In DeepSoluE, prediction features were combined by the 100D features optimized from the five physicochemical feature descriptors using a genetic algorithm, 100D embedded semantic features, and 19D features related to sequence identity and special physicochemical characters (refer to Methods for details). The combined 219D features were fed into the DeepSoluE architecture. To avoid overfitting, an early stopping strategy based on the validation loss is applied when training the LSTM model. Then, the model was validated. Independent test data were used to test the model that showed the best performance on the validation data. As shown in Table 1, the training and validation processes are measured on the metric ACC, and the independent testing results are measured on all the five metrics. For the ten trained models, the maximum ACC was achieved on Model 6 (0.6574), and the minimum ACC was obtained on Model 4 (0.6386). Then, the trained models were evaluated on the independent test dataset. Model 1 resulted in the best MCC (0.2101) and AUC (0.6254), while Model 5 ranked last on MCC (0.1931) and AUC (0.6163). For the sake of convenience and comparison, the average values of the ten models were used to measure the performance of DeepSoluE. Based on that, DeepSoluE achieved an average training ACC of 0.6463 and an average validation ACC of 0.6401. On independent test data, DeepSoluE achieved an average ACC of 0.5885, SN of 0.6108, SP of 0.5661, MCC of 0.1776, and AUC of 0.6197.

**Table 1:** Individual and ensemble model performance on training and testing data

Model	Training ACC	Validation ACC	Test ACC	Test MCC	Test AUC
Model 1	0.6477	0.6407	0.6006	0.2014	**0.6254**
Model 2	0.6456	0.6434	0.5903	0.1821	**0.6195**
Model 3	0.6394	0.6381	0.5861	0.1724	**0.6223**
Model 4	0.6386	0.6329	0.5723	0.1480	**0.6184**
Model 5	0.6550	0.6521	0.5858	0.1717	**0.6163**
Model 6	0.6574	0.6442	0.5932	0.1865	**0.6168**
Model 7	0.6494	0.6203	0.5952	0.1905	**0.6191**
Model 8	0.6437	0.6317	0.5858	0.1727	**0.6205**
Model 9	0.6404	0.6640	0.5877	0.1756	**0.6201**
Model 10	0.6457	0.6334	0.5874	0.1749	**0.6189**
Average^a^	0.6463	0.6401	0.5885	0.1776	**0.6197**
Ensemble^b^	-	-	**0.5952**	**0.1904**	**0.6259**

^a^ Metrics average value for Model 1 to Model 10, ^b^ metrics value for the ensemble model

As described in methods, each trained model takes 9 of the ten folds of the complete training dataset as input. To give full play to the advantages of ensemble learning, an ensemble method (soft voting, threshold = 0.4) is applied to build an integration model. As shown in Table 1, the integrated model achieved better performance than the individual model, indicating that the ensemble strategy is effective for model performance improvement.

**Fig. 2:**
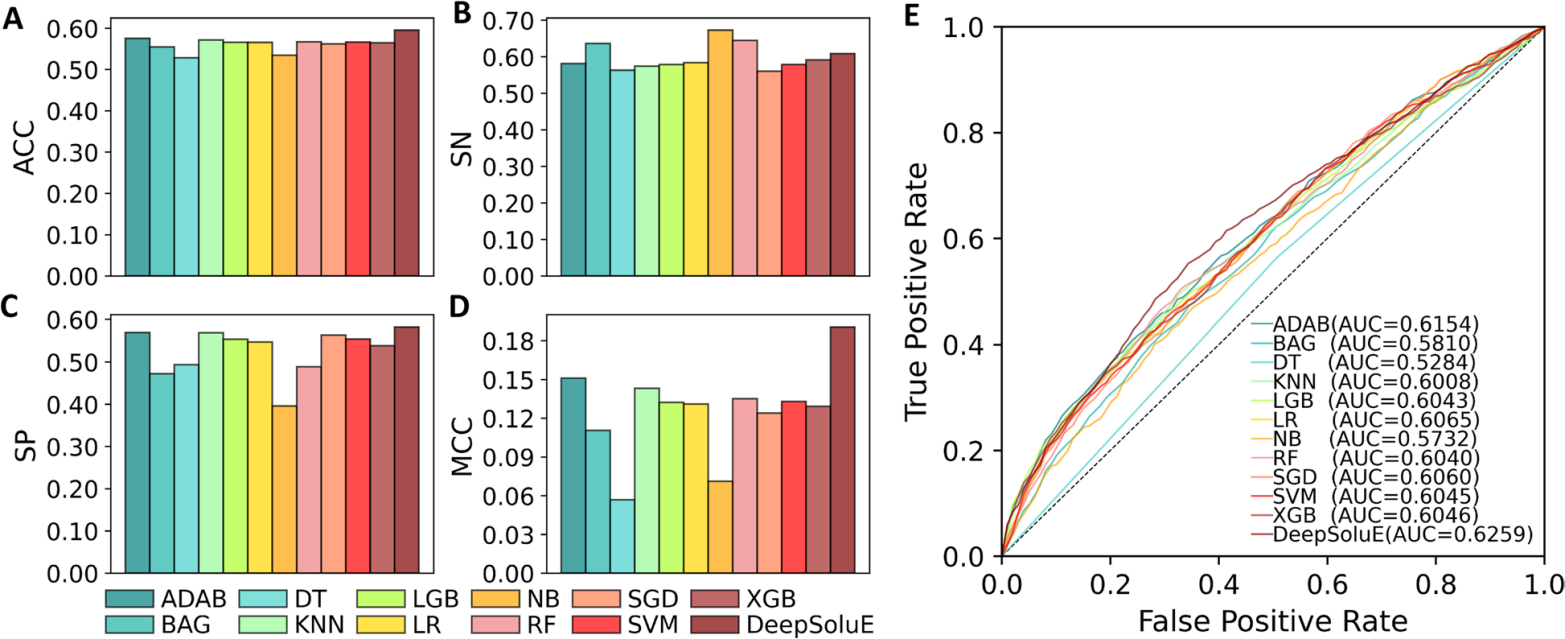
Performance comparison of DeepSoluE and 11 conventional machine learning methods

To further assess the efficacy of the LSTM architecture, we compared DeepSoluE with 11 popular traditional machine learning algorithms, including the AdaBoost classifier (ADAB), bagging (BAG), decision tree (DT), k-nearest neighbor (KNN), light gradient boosting machine (LGB), logistic regression (LR), naïve Bayesian (NB), random forest (RF), stochastic gradient descent (SGD), support vector machine (SVM) and extreme gradient boosting (XGB) algorithms. Each of the 11 models is trained on the training dataset and evaluated on the independent test dataset (refer to Additional file 1: Table S3 for model hyperparameter optimization). Figure 2 presents the values of the five metrics, in which DeepSoluE outperformed all the rest of the classifiers in terms of ACC, SP, MCC, and AUC (Additional file 3: Table S4). For metric SN, the highest value is obtained on the NB classifier, followed by the RF and BAG classifiers. DeepSoluE ranked fourth among the 12 models. It is worth noting that DeepSoluE achieved more balanced performance with |SN-SP|=2.65%, while the NB classifier returned |SN-SP|=27.74%, the RF classifier returned |SN-SP|=15.68%, and the BAG classifier returned |SN-SP|=16.45%. As indicated in Eqs. (2) and (3), SN and SP actually constrain each other as they measure a predictor from two different angles [21, 34]. Maintaining a balance between SN and SP is crucial for an accurate model to provide an unbiased prediction. Overall, these results demonstrate that DeepSoluE is significantly superior and more robust than the traditional classifiers.

### Feature contribution and dependency analysis

**Fig. 3:**
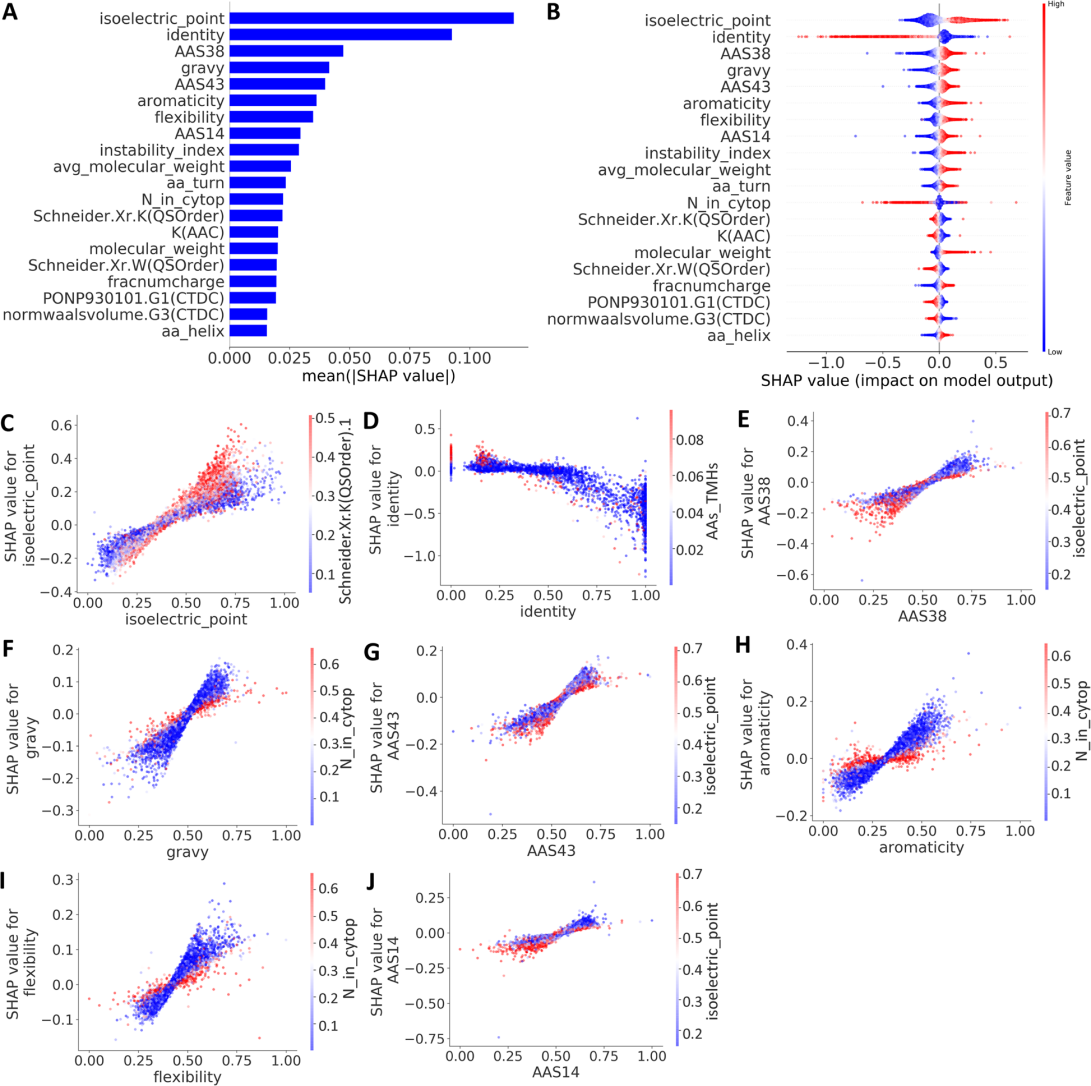
Feature contribution and dependency analysis. **A** The 20 most important features. **B** Summary plot for SHAP values. For each feature, one point corresponds to a single sample. The SHAP value along the *x*-axis represents the impact that feature had on the model’s output for that specific sample. Features in the higher position in the plot indicate the more important it is for the model. **C**–**J** SHAP dependence plots. These plots show the effect that a single feature has on the model predictions and the interaction effects across features. Each point corresponds to an individual sample, the value along the *x*-axis corresponds to the feature value, and the color represents the value of the interacting feature

SHapley Additive exPlanation (SHAP) values [17] were applied to infer informative features of DeepSoluE. First, the top 20 most important features are calculated and depicted by the SHAP summary plot. As shown in Fig. 3A, physicochemical properties critical for protein solubility include protein isoelectric point, gravy, aromaticity, flexibility, instability index, molecular weight, and fraction charge. Protein structure and motifs/patterns that are related to protein solution are composed of an aa turn, an aa helix, and lysine (K), a polar amino acid group (“KPDESNQT”) that is defined by hydrophobicity attribute PONP930101 and amino acids (“MHKFRYW”) that have a larger residual volume according to the definition of normalized van der Waals volume [18].

Furthermore, how the feature values affect the model prediction was explored. Figure 3B shows their corresponding summary plots of the top 20 most important features, which illustrates how high and low feature values were related to the model output. For example, high values of isoelectric point are associated with positive impacts on protein solution, while low values have negative impacts. Similar feature value influences are also observed in the other 12 of the top 20 features. Opposite changing trends are observed by the other 7 features; i.e., high values of identity weaken the model behavior, and low values of identity boost model performance (Fig. 3B). In addition, SHAP values of several decisive features, e.g., isoelectric point (−0.4 to 0.6), identity (−1.25 to 0.75), AAS38 (−0.6 to 0.4), vary in a larger range than others, which suggests why they dominate the model’s behavior; the reverse situation explains why the aa helix (−0.15 to 0.1) and norm Waals volume. G3(CTDC) (−0.15 to 0.1) is less important than the others since changes in its value result in less influence on the corresponding SHAP values.

From the biological aspect, the pH of the solution affects the nature and the distribution of the protein’s net charge, and, generally, protein exhibits the least solubility at the isoelectric point [19]. Therefore, proteins with a higher isoelectric point have a net negative or positive charge, and interact with more water, which may partly explain why isoelectric point was the most important feature and high value has a positive impact on protein solubility (Fig. 3AB). Similar, feature aa_turn related to three hydrophilic amino acids (G, N, and S), amino acids that have a larger flexibility present a larger contact area with the solvent, both of them contributed to protein solubility. Protein sequence that enriched with charged amino acids (R, K, D, E) are also beneficial for their solubility. Notably, several opposite situations are observed on our results, for example, higher polar amino acid content (PONP930101) showed a negative impact on protein solubility (Fig. 3B), this implies factors that influence protein solubility is far from clear and further study on this area is necessary.

Finally, SHAP dependence plots were used to provide meaningful insights into interaction effects across features. The dependence plots of the top 20 features are shown in Fig. 3C–J and Additional file 2: Fig. S2. Feature turning points can be visualized; for example, the proposed DeepSoluE takes approximately 0.4 as a turning point for the feature isoelectric point, and the feature values higher than that value contribute to performance boost (Fig. 3C). The turn point of feature identity is approximately 0.5, and values higher that value change SHAP values from negative to positive. For feature interactions, Fig. 3D shows that high identity values (range 0.4 to 1.0) with low AAs_TMHs values (0.1–0.2) have a negative impact on model behavior (SHAP values<0), while low values of AAs_TMHs show little impact. A high feature value of aromaticity (0.0–0.3) with a low feature value of N_in_cytop contributes to accurate model prediction, while a low feature value of N_in_cytop has the opposite effect. Similar feature interaction patterns were observed in two other feature pairs (Fig. 3H and I). More feature interaction patterns can be seen in Fig. 3C–J and Additional file 2: Fig. S2.

### Comparison with existing predictors

The independent testing set of this study is used to evaluate and compare DeepSoluE with 12 previously published tools. The recommended parameters, such as the model decision threshold (T), of each tool are adopted for result evaluation. Table 2 provides details of the comparative analysis results. DeepSoluE exhibited the best performance when evaluated by the metrics ACC and MCC. Although the best SN and SP were achieved by the SWI and DeepSol models, respectively, the prediction results of the two models are seriously biased. SWI achieved an SN of 0.7781; however, the SP of this model was 0.3400, which resulted in |SN −SP|=43.81%. This finding suggests that SWI tends to predict a query protein as soluble. Similarly, DeepSol resulted in |SN −SP|=76.06%, which means that the prediction result of DeepSol is heavily skewed toward insoluble.

**Table 2:** Performance comparison of DeepSoluE with existing predictors in protein solubility prediction on independent test data

Method	T	ACC	SN	SP	|SN-SP|%	MCC	TP	TN	FP	FN
RPSP	0.5	0.4980	0.3232	0.6735	35.0323	0.0000	501	1044	506	1049
ccSOL omics	0.5	0.5080	0.5703	0.4452	12.5161	0.0200	884	690	860	666
SKADE	0.5	0.4920	0.1026	0.8813	77.8710	-0.0300	159	1366	184	1391
SOLpro	0.5	0.5200	0.4219	0.6187	19.6774	0.0400	654	959	591	896
Protein-Sol	0.5	0.5160	0.6813	0.3510	33.0323	0.0300	1056	544	1006	494
DeepSol	0.5	0.5290	0.1484	**0.9090**	76.0645	0.0900	230	1409	141	1320
rWH	0.5	0.5400	0.4323	0.6484	21.6129	0.0800	670	1005	545	880
ESPRESSO	0.5	0.5380	0.6471	0.4284	21.8710	0.0800	1003	664	886	547
CamSol	1	0.5410	0.4361	0.6458	20.9677	0.0800	676	1001	549	874
SWI	0.5	0.5590	**0.7781**	0.3400	43.8065	0.1300	1206	527	1023	344
PROSO II	0.6	0.5800	0.4065	0.7529	34.6452	0.1700	630	1167	383	920
SoluProt	0.5	0.5850	0.6058	0.5632	4.25810	0.1700	939	873	677	611
DeepSoluE	0.4	**0.5952**	0.6084	0.5819	**2.64520**	**0.1904**	943	902	648	607

Performance values of most methods are adopted from [11]

To further make a reasonable comparison, models that presented a |SN–SP| < 20% were filtered for further analysis. Based on the preconditions, four models, ccSOL omics, SOLpro, SoluProt, and DeepSoluE, are retained. Among them, DeepSoluE shows the greatest value on metrics ACC, TP, TN, SN, SP, and MCC, followed by SoluProt and SOLpro. Notably, while the datasets of DeepSoluE are homology reduced to 25% and the testing set is independent of the training set, other tools’ training sets might have a high sequence overlap with our test set. For example, the DeepSol and SKADE training sets presented a 74% overlap with our testing set, and SoLpro had an overlap of 15.5%. More information on the sequence identity of previous tools is presented in [11]. Of note, the model with high sequence redundancy between its training set and our testing set will benefit from the comparison results, as listed in Table 2. In conclusion, all these results demonstrate that DeepSoluE outperformed the existing prediction algorithms for protein solubility prediction.

## Conclusions

In this study, a deep learning predictor called DeepSoluE was developed to accurately predict protein solubility in *E. coli*. The hybrid features composed of physicochemical patterns and semantic information were used to represent sequence patterns. As a result, DeepSoluE outperforms the existing predictors for solubility prediction and achieves a more balanced performance. Furthermore, SHAP values were employed for model explanation and investigation of the impact of specific features on the model predictions and their interaction effects. Although the proposed model achieves performance improvement, the accuracy of the currently available predictors is still less than 60%, and there is still room for further improvement by using more advanced algorithms and incorporating more informative heterogeneous features. For example, using the protein 3D structure information is a possible direction to further improve our work as the 3D structure provides more geometric information of each amino acid residual, and several neural network-based methods, such as Alphafold2 [20] and RGN2 [22], can generate the predicted 3D structure information of proteins. For convenience, a user-friendly web server has been made publicly available to implement DeepSoluE. We expect that DeepSoluE can be complementary to hands-on experiments and facilitate our understanding of protein function.

## Methods

**Fig. 4:**
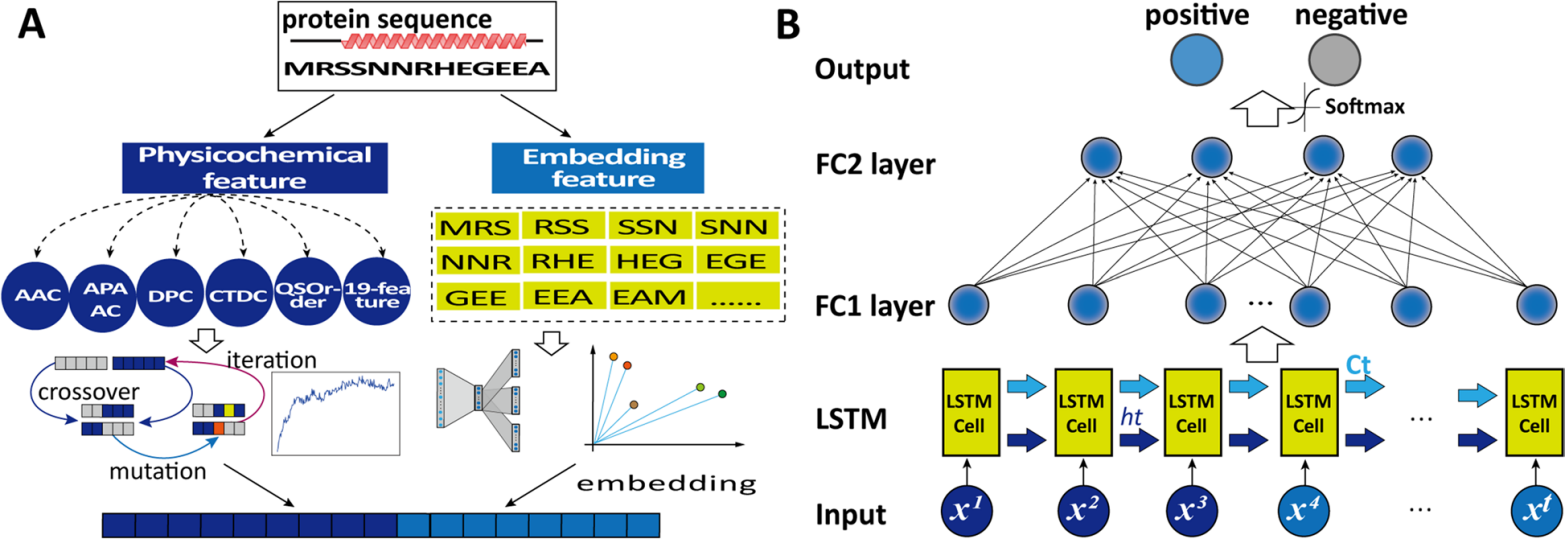
The DeepSoluE workflow. **A** Physicochemical feature encoding, feature optimization, and distributed representation of protein sequences. **B** Neural network architectures of DeepSoluE; FC, fully connected layer

Figure 4 illustrates the workflow of constructing the DeepSoluE model, which includes three main steps: (i) sequence preprocessing, (ii) sequence physicochemical feature extraction, distributed representation, and feature dimensionality reduction, and (iii) feature combination, neural network training and evaluation. More details regarding each step are described below.

### Benchmarking datasets

Several benchmark datasets with different sequence numbers and identities have been used for protein solubility modeling (Additional file 1: Table S1). As listed in Table S1, five of the 13 models, rWH, CCSOL [23], ESPRESSO [24], CamSol [25], and Protein-Sol [26], were built on datasets extracted from specifically published research, and the five datasets were not considered for model training in our study because of their insufficient representation. The remaining eight predictors were built based on three comprehensive databases, namely, TargetDB [27], PepcDB [27], and TargetTrack [28]. TargetDB collects protein target information from nine NIH Protein Structure Initiative (PSI) centers and ten other international structural genomics sites [27]. PepcDB is an extension of TargetDB and enriches the record information, such as historical status and experimental details, for each trial [29]. TargetTrack, the latest and most widely used dataset, is merged by TargetDB and PepcDB [28]. This database integrates information from the PSI project and contains information related to almost 300 thousand unique protein targets with the effort of dozens of structural genomics centers across the world [30, 31]. Therefore, the filtered TargetTrack database adopted in SoluProt [11] was used as the training dataset in this study. The original TargetTrack database was cleaned by several stringent filtering rules; see [11] for more details, and the sequence identity was reduced to 25%. Finally, 11436 proteins (5718 soluble vs. 5718 insoluble) were used for model training. The SoluProt test data collected from the North East Structural Consortium (NESG) were applied for model-independent testing. The original NESG was filtered using the same procedure as the training set, and sequences from the test set that had a global sequence identity above 25% with the training set were removed [11]. Finally, 3100 proteins (1550 soluble vs. 1550 insoluble) were retained for model independent testing and comparison.

### Feature representation

Extracting strong discriminative features is crucial for building a reliable and superior model. In this study, two groups of feature-encoding algorithms were used to represent the protein sequences.

#### Sequence physicochemical-based features

Five physicochemical feature descriptors were employed to formulate the protein sequences [18]. These features are amino acid composition (AAC), amphiphilic pseudoamino acid composition (APAAC), di-peptide composition (DPC), composition (CTDC), and quasi-sequence-order (QSOrder). They are described in detail in the Additional file 4. A brief introduction of these methods is as follows. AAC calculates the frequencies of all 20 amino acids in a protein sequence [32, 33]. APAAC incorporates partial sequence-order effects and correlation functions by using the hydrophobicity and hydrophilicity properties of the constituent amino acids in a protein [35]. DPC computes the frequencies of all dipeptides [36]. CTDC calculates the transition frequencies of three kinds of residue pairs that are categorized by their physicochemical properties [37], and thirteen types of physicochemical properties are used for CTDC (Additional file 5: Table S5). QSOrder encodes the sequence order based on the Schneider–Wrede physicochemical distance matrix [38] and the Grantham chemical distance matrix [39]. In addition, nineteen physicochemical features calculated by Biopython (15 features) [40], TMHMM (3 features) [41], and USEARCH (1 feature) [42] were also used for sequence formulation (Additional file 5: Table S6) [43–46].

#### Word embedding-based features

Word embedding techniques such as one hot encoding have been widely used in the bioinformatics field. Recently, several efficient word embedding algorithms, e.g., word2vec [47], were proposed for distributed representation of all kinds of biological sequences, such as proteins [48, 49], DNA [50], mRNA [51, 52], noncoding RNA [53, 54], and 16S/18S rRNA [55, 56]. In the framework of word2vec, each word from a vocabulary is characterized by its context and represented as a predefined n-dimensional numeric vector, where similar words have close vectors (Fig. 4A). The process is briefly described as follows. First, a protein sequence with n amino acids was regarded as a sentence, and the biocorpus was obtained in an overlapping manner by moving a window of size *k* (*k* < *n*) along the sequence with a stride length of 1. Given this biocorpus, each word was embedded into a fixed N-dimensional numeric vector using word2vec with a skip-gram model that attempts to predict the context words from the focus word. Thus, each word was presented as a numeric vector of size *N*, and each sequence was represented by the average of all corpora in the sequence, which is a vector of size N [52]. We used the Gensim library (https://radimrehurek.com/gensim/) to create a word2vec representation for the protein samples.

### Feature selection using a genetic algorithm

The above five physicochemical-based descriptors generate a feature subset with 523D. Using all the features for model training may cause information redundancy, which, in turn, influences model performance and increases computing complexity and time. Hence, a genetic algorithm [57, 58] was employed to choose the optimal feature subsets from the original 523D features. The process is briefly described as follows. First, a genetic algorithm begins with a constant number of populations (chromosomes), namely, feature subsets, as ancestors. In this study, the number of chromosomes was set to 200, and the gene number of each chromosome, i.e., the feature dimension of the feature subset, was set to 100. During each iteration (generation), each chromosome is evaluated with a specified fitness function to maximize classification accuracy. Then, three genetic operators, selection, crossover, and mutation, are used to generate new populations (offspring) (Fig. 4A). A stochastic tournament selection operator was adopted to probabilistically select individuals from a population as parents for later breeding. A two-point crossover operation was performed to create offspring, and each individual had a probability of 0.0003 to mutate. The generation time is set to 500. For each generation, offspring will inherit the favorable characteristics of their parents.

### Neural network architectures

The optimized physicochemical features and the embedding features were concatenated into a vector and then fed into the LSTM network [59] for model construction and evaluation. We used TensorFlow v2.4 to implement the LSTM model. The main architecture of the network consisted of one LSTM layer and three fully connected layers (Fig. 4B). The combined features were first fed into the LSTM layer to extract potential feature patterns and capture the short-term and long-term order dependencies among features. The output of the last LSTM cell served as the input of three fully connected layers. A dropout layer was connected before the last fully connected layer. The ReLU function was used in the first two fully connected layers, and the softmax function was used for binary classification in the final output layer. During learning, four hyperparameters (the number of units of the LSTM layer, the number of units in the two fully connected layers, and the learning rate) were optimized. A Kerastuner library (https://keras.io/keras_tuner/) was used to automatically turn the hyperparameters, as listed in Additional file 5: Table S7.

### Model training and evaluation

The entire training dataset contains 11436 proteins (5718 soluble vs. 5718 insoluble). It was divided into 10 folds, namely, Fold 1,…, Fold 10, using stratified sampling. Based on this split, 10 LSTM models, denoted as Model 1,…, Model 10, were constructed. For Model k, Fold k acts as a validation set, and the remaining 9 folds act as the training sets. The training set was used to fit the model with the optimal parameters listed in Additional file 5: Table S7. The validation set was used to validate the performance of the model with the most suitable parameters. Finally, the independent testing dataset was used to provide an unbiased performance evaluation of the final model.

Five metrics were used to comprehensively measure the performance of the ensemble model: ACC, specificity (SP), sensitivity (SN), Matthews correlation coefficient (MCC), and area under curve (AUC). They were calculated as follows:

1 $$\textrm{ACC}=\frac{TP+ TN}{TP+ TN+ FP+ FN}$$

2 $$\textrm{SN}=\frac{TP}{TP+ FN}$$

3 $$\textrm{SP}=\frac{TN}{TN+ FP}$$

4 $$\textrm{MCC}=\frac{TP\times TN- FP\times FN}{\sqrt{\left( FP+ TP\right)\left( FN+ TP\right)\left( FP+ TN\right)\left( FN+ TN\right)}}$$

The metric AUC calculates the area under the receiver operating characteristic curve based on the false-positive rate (FPR) and the true positive rate (TPR) under various thresholds. The TPR and the FPR were calculated as follows:

5 $$\textrm{TPR}=\frac{TP}{TP+ FN}$$

6 $$\textrm{FPR}=\frac{FP}{TN+ FP}$$

where TP = true positive, FP = false-positive, TN = true negative, and FN = false negative. SN and SP were employed to evaluate the model performance with respect to the positive and negative samples, respectively. The remaining three metrics are global prediction performance indicators.

## Supplementary Information

**Additional file 1: Table S1.** Computational approaches for predicting protein solubility (sorted by published year). **Table S2.** Descriptor parameter search range and the best values. **Table S3.** Hyperparameters search range for the 11 traditional classifiers.

**Additional file 2: Figure S1.** The heatmap shows the accuracy values of the model constructed with different k (length of k-mer) and w (window size) values. **Figure S2.** The SHAP dependence plots. These plots show the effect that a single feature has on the models predictions and the interaction effects across features. Each point corresponds to an individual sample, the value along the x axis corresponds to feature value, the color represents the value of the interacting feature.

**Additional file 3: Table S4.** Performance comparison of DeepSoluE and 11 conventional machine learning methods.

**Additional file 4:.** Sequence physicochemical-based features.

**Additional file 5: Table S5.** Thirteen types of physicochemical properties that used for computing the features of CTDC. **Table S6.** 15 physicochemical features calculated by Biopython and three features from TMHMM. **Table S7.** Hyperparameters for LSTM model.

## Data Availability

All code and data generated or analyzed during this study are included in this published article, its supplementary information files, and publicly available repositories. Which are available in the Zenodo repository (https://zenodo.org/record/7418334) and GitHub (https://github.com/wangchao-malab/DeepSoluE/).

## Abbreviations

ACC: Accuracy
ADAB: adaBoost classifier
AAC: Amino acid composition
APAAC: Amphiphilic pseudoamino acid composition
AUC: Area under curve
BAG: Bagging
CTDC: Composition
DT: Decision tree
DPC: Di-peptide composition
*E. coli*: *Escherichia coli*
XGB: Extreme gradient boosting
FPR: False-positive rate
KNN: k-nearest neighbor
LSTM: Long-short-term memory
LGB: Light gradient boosting machine
LGB: Light gradient boosting machine
LR: Logistic regression
MCC: Matthews correlation coefficient
NB: Naïve Bayesian
QSOrder: Quasi-sequence-order
RF: Random forest
SN: Sensitivity
SFS: Sequential forward search
SHAP: SHapley Additive exPlanation
SP: Specificity
SGD: Stochastic gradient descent
SVM: Support vector machine
TPR: True-positive rate
