# Supplementary material for: Prediction of protein solubility based on sequence physicochemical patterns and distributed representation information with DeepSoluE
Source: BMC Biol. 2023 Jan 24;21:12. doi: 10.1186/s12915-023-01510-8 (PMC9875434; doi:10.1186/s12915-023-01510-8)
Supplement: Supplementary file 1 — Additional file 1: Table S1. Computational approaches for predicting protein solubility (sorted by published year). Table S2. Descriptor parameter search range and the best values. Table S3. Hyperparameters search range for the 11 traditional classifiers. [file 12915_2023_1510_MOESM1_ESM.docx]

**Table S1** Computational approaches for predicting protein solubility (sorted by published year)

| **Method** | **Feature information** | **Prediction algorithm** | **Website/source code available** | **Database/sequence homology/independent test data** | **Reference** |
| --- | --- | --- | --- | --- | --- |
| rWH | (1) turning forming residue fraction (Asn, Gly, Pro and Ser);  (2) approximate-charge average (relative numbers of Asp plus Glu vs. Lys plus Arg) | Statistical analysis | A model updated in 2009 is available https://www.biotech.ou.edu/, which is logistic regression of 32 possible parameters based on 212 proteins. | 81 proteins analysis by Davis et al., 1999; Based on that, Wilkinson and Harrison, 1991 used five amino acid-based parameters to predict protein solubility. | Davis et al., 1999; Wilkinson and Harrison, 1991 |
| PROSO | (1) AAC; (2) DPC; (3) TPC; (4) AA index including eight numeric scales | SVM, NB | Web server provided but currently unavailable:  (http://webclu.bio.wzw.tum.de:8080/proso) | TargetDB database  Sequence redundancy was reduced by CD-HIT (50%)  10-fold stratified cross-validation  No independent test data were used | Smialowski et al., 2007 |
| SOLpro | (1-3) AAC, DPC and TPC (4-21) AAC, DPC and TP in six reduced alphabets;  (22) Sequence length; (23) Turn-forming residues fraction; (24) Absolute charge per residue; (25) Molecular weight; (26) GRAVY index; (27) Aliphatic index; (28) Beta residues fraction; (29) Alpha residues fraction; (30) Number of domains; (31) Exposed residues fraction | SVM | Webserver and source code available at:  http://scratch.proteomics.ics.uci.edu/ | PDB soluble proteins +SwissProt enzymes + TargetDB databases + ITB  Sequence redundancy was reduced by BLASTCLUST (25% similarity). Finally, 17 408 proteins (8704 soluble and  8704 insoluble) were used for model training.  10-fold cross-validation  No independent test data were used | Magnan et al., 2009 |
| PROSO II | (1) DPC; (2) sequence similarity calculated by Parzen window model. | Logistic regression | Web server provided but currently unavailable:  (http://mips.helmholtz-muenchen.de/prosoII) | pepcDB database including information Protein Structure Initiative centers and TargetDB database. pepcDB database is the predecessor of TargetTrack database  Sequence redundancy was reduced by CD-HIT (90%). Finally, 82999 proteins were retained.  10-fold cross-validation  A holdout set including 1764 proteins was used for independent test | Smialowski et al., 2012 |
| CCSOL | (1) α-helix; (2) β-sheet; (3) β-turn; (4) disorder/coil; (5) hydrophobicity/ hydrophilicity; (6) AAC; (7) polarity; (8) buried residues; (9) mutability; (9) transmembrane tendency | SVM | http://s.tartaglialab.com/page/ccsol_group | 3043 proteins collected from literature Niwa et al. (2009).  Leave-one-out cross-validation  No independent test data were used | Agostini et al., 2012 |
| ccSOL omics | (1)coil/disorder; (2)hydrophobicity; (3) hydrophilicity; (4)β-sheet; (5)α-helix; | Fourier’s coefficients | http://s.tartaglialab.com/page/ccsol_group | TargetTrack database 36990 with 30% redundancy (CD-HIT)  10-fold cross-validation  three independent datasets were used for model test | Agostini et al., 2014 |
| ESPRESSO | (1) chemical property group:  aliphatic, aromatic, hydroxyl, acidic…  (2) sequence patterm group:  hydrophobic; polar; small; proline; tiny; aliphatic;aromatic; positive; negative; charge | SVM | Web server provided but currently unavailable:  http://mbs.cbrc.jp/ESPRESSO | Data is collected from literature Hirose et al., 2011.  Sequence redundancy was reduced by and CD-HIT and ALIGN0 (25%)  10-fold cross validation | Hirose and Noguchi, 2013 |
| CamSol | (1) hydrophobicity; (2) the charge (at pH 7); (3) the α-helix propensity; (4) and the β-strand propensity; | Linear and logistic regression | http://www-vendruscolo.ch.cam.ac.uk/camsolmethod.html | Data is collected from literature Wilkinson et al., 1991, Magnan et al., 2009, Smialowski et al., 2012, Agostini et al., 2012.  56 protein variants were used for test | Sormanni et al., 2015 |
| Protein-Sol | (1) AAC; (2) 7 specific amino acid composition; (3) sequence length; (4) pI; (5) hydropathy; (6) absolute charge at pH 7; (7) fold propensity; (8) disorder; (9) sequence entropy; (10) β-strand propensity | Linear model | http://protein-sol.man  chester.ac.uk | 2395 proteins were used for model training  Three independent datasets were used for model test | Hebditch et al.,  2017 |
| Deep-Sol | (1) One hot; (2) sequence length; (3) molecular weight; (4) absolute charge; (5) aliphatic indices (AIs); (6) GRAVY; (7) fraction of turn-forming residues; (8) three-and eight-state SS; (9) fraction of exposed residues; (10) multiplied the FER by the hydrophobicity indices | CNN | Source code at:  https://doi.org/10.5281/zenodo.1162886 | Dataset was collected from PROSO II, namely pepcDB database  Sequence redundancy was reduced by CD-HIT (90%). Finally,  28972 soluble vs. 40 448 insoluble proteins were used for model train  Independent test set containing 1000 soluble and 1001 insoluble proteins was used for model test | Sameer et al., 2017 |
| SKADE | Embedding feature | Neural Network | Source code at:  https://bitbucket.org/eddiewrc/skade/src | Dataset was collected from Deep-Sol, including 28972 soluble and 40448 insoluble proteins  Independent test set: 1000 soluble vs. 1001 insoluble | Daniele et al., 2020 |
| SWI | (1)isoelectric point; (2) turn, (3) aromaticity; (4) helix; (5) molecular weight; (6) gravy; (7) flexibility; (8) instability index; (9) miscellaneous protein sequence properties | Arithmetic mean | Source code at https://github.com/Gardner-BinfLab/SoDoPE_paper_2020  Web server at:  https://tisigner. com/sodope | PSI: Biology data (8238 soluble vs. 3978 insoluble)  independent test set: eSOL (1934 soluble vs. 1264 insoluble)  10-fold cross validation | Bikash et al., 2020 |
| SoluProt | (1) AAC, (2) ADC; (3) fraction of charged amino acids (R, K, D, E); (4) ratio of K and R content; (5) fraction of helix amino acids (V, I, Y, F, W, L); (6) fraction of sheet amino acids (E, M, A, L); (7) fraction of turn amino acids (N, P, G, S); (8) molecular weight; (9) Molecular weight; (10) fraction of aromatic amino acids (Y, W, F); (11) flexibility; (12) grand average of hydropathy; (13) isoelectric point; (14) instability index; (15) average flexibility; (16) secondary structure content; (17) average disorder; (18) content of amino acids in transmembrane helices; (19) maximum identity to the *E.coli* PDB subset | GBM | https://loschmidt.chemi.muni.cz/soluprot/ | TargetTrack database  Sequence redundancy was reduced by MMseqs2 (25% identity). Finally, 5718 soluble and 5718 insoluble were used for model train  Independent test set: NESG (1550 soluble vs. 1550 insoluble) | Jiri et al., 2021 |

**Table S2** Descriptor parameter search range and the best values

| **Feature encodings** | **Parameters** | **Search range** | **Optimal value** |
| --- | --- | --- | --- |
| QSorder | nlag | [1,2,3,…,10] | 1 |
| APAAC | λ | [1,2,3,…,10] | 1 |

**Table S3** Hyperparameters search range for the 11 traditional classifiers.

| **Method** | **Parameter grid search ranges** |
| --- | --- |
| Random Forest (RF) | n_estimators: [10, 20, …, 200]  max_features: [1, 2, 3, …,10]  min_samples_split: [1, 2, 3, …,10] |
| k-nearest neighbor (KNN) | n_neighbors: [1, 2, 3, …, 100] |
| Support Vector Machine (SVM) | C: [0.01, 0.05, 0.1, 0.5, 1, 5, 10, …, 100]  gamma: [0.0001, 0.0002, 0.0004, …, 0.1, 0.2, 0.4, …, 8]  Kernel: [rbf] |
| naive Bayesian (NB) | - |
| light gradient boosting machine (LGB) | n_estimators: [10, 20, …, 200]  learning_rate: [0.01, 0.05, 0.1]  max_depth: [2, 4, 6, 8]  num_leaves: [10, 20, 30, …, 100] |
| AdaBoost classifier (ADAB) | n_estimators: [10, 20, …, 200]  learning_rate: [0.05, 0.1, 0.15, 0.2, …, 0.95, 1.0] |
| Bagging (BAG) | n_estimators: range: (10,210,10),  max_features: range(1,11,1) |
| Logistic regression (LR) | - |
| Decision tree (DT) | max_features: range (1,11,1),  min_samples_split: range(1,11) |
| Stochastic gradient descent (SGD) | loss: ['hinge', 'log', 'modified_huber', 'squared_hinge','perceptron'],  penalty: ['none', 'l2', 'l1','elasticnet'] |
| Extreme gradient boosting (XGB) | n_estimators: range (10,110,10),  learning_rate: [0.01,0.020,0.03,0.04,0.05,0.06,0.07,0.08,0.09,0.1],  max_depth: range(1,20,2) |
