# Supplementary material for: Prediction of protein solubility based on sequence physicochemical patterns and distributed representation information with DeepSoluE
Source: BMC Biol. 2023 Jan 24;21:12. doi: 10.1186/s12915-023-01510-8 (PMC9875434; doi:10.1186/s12915-023-01510-8)
Supplement: Supplementary file 2 — Additional file 2: Figure S1. The heatmap shows the accuracy values of the model constructed with different k (length of k-mer) and w (window size) values. Figure S2. The SHAP dependence plots. These plots show the effect that a single feature has on the models predictions and the interaction effects across features. Each point corresponds to an individual sample, the value along the x axis corresponds to feature value, the color represents the value of the interacting feature. [file 12915_2023_1510_MOESM2_ESM.docx]

**
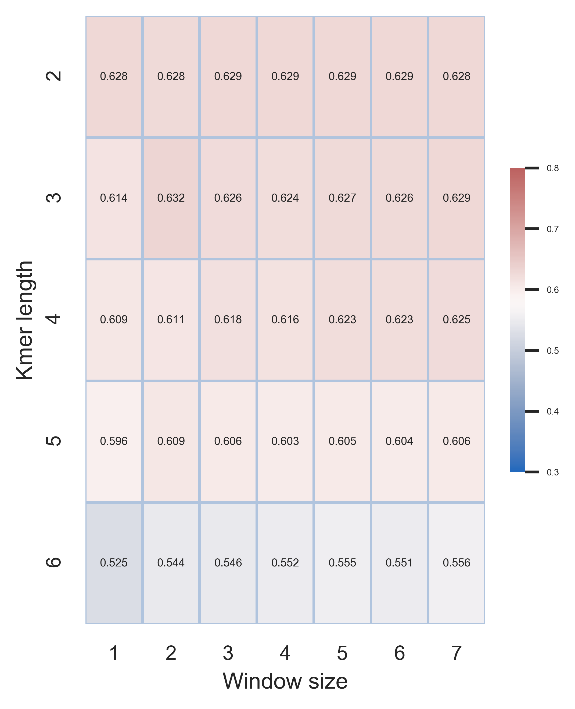
**

**Figure S1** The heatmap shows the accuracy values of the model constructed with different k (length of k-mer) and w (window size) values

**
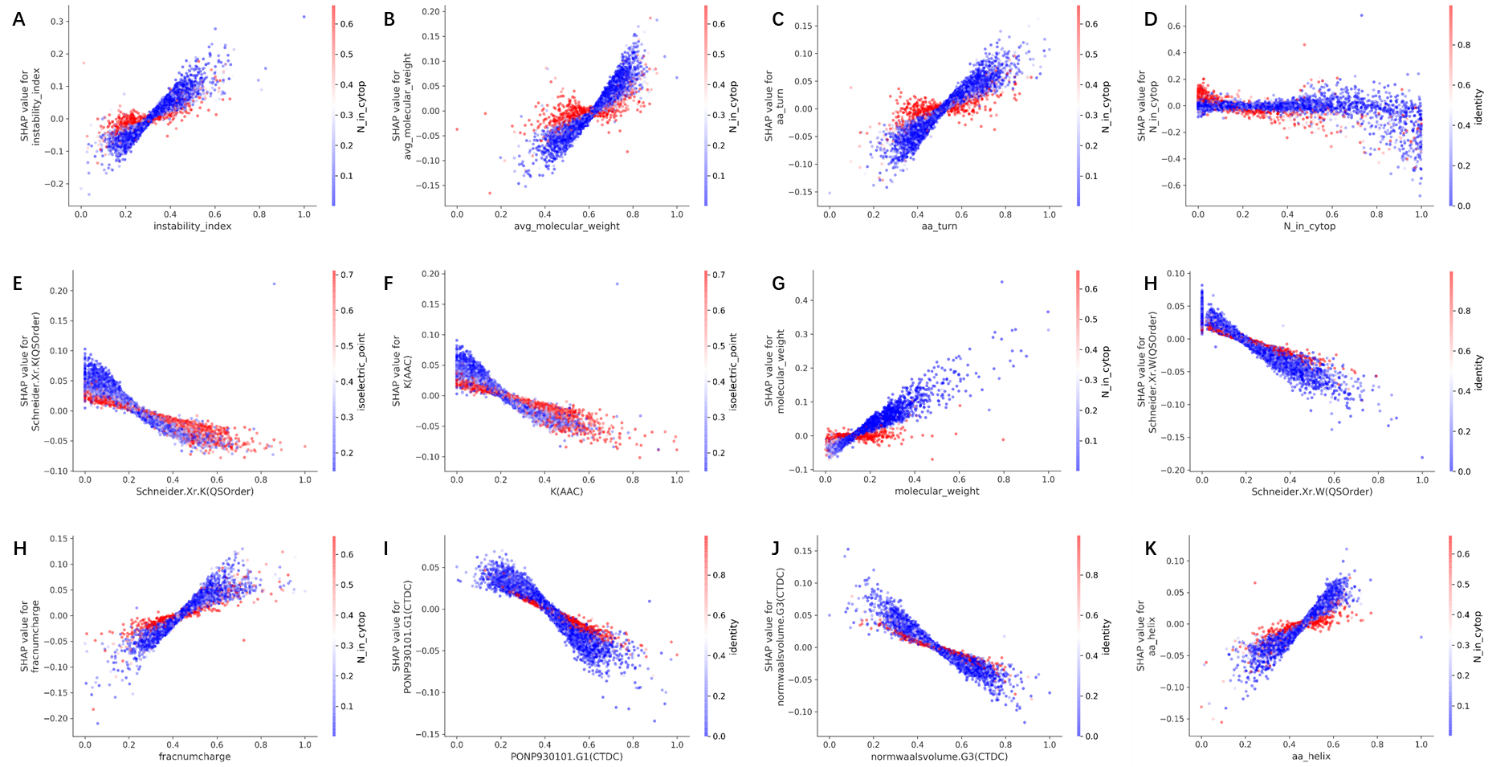
**

**Figure S2** The SHAP dependence plots. These plots show the effect that a single feature has on the models predictions and the interaction effects across features. Each point corresponds to an individual sample, the value along the x axis corresponds to feature value, the color represents the value of the interacting feature.
