# Supplementary material for: Prediction of protein solubility based on sequence physicochemical patterns and distributed representation information with DeepSoluE
Source: BMC Biol. 2023 Jan 24;21:12. doi: 10.1186/s12915-023-01510-8 (PMC9875434; doi:10.1186/s12915-023-01510-8)
Supplement: Supplementary file 4 — Additional file 4:. Sequence physicochemical-based features. [file 12915_2023_1510_MOESM4_ESM.docx]

**Sequence physicochemical-based features**

Five physicochemical feature descriptors were employed to formulate the protein sequences, including amino acid composition (AAC), amphiphilic pseudo-amino acid composition (APAAC), di-peptide composition (DPC), composition (CTDC) and quasi-sequence-order (QSOrder).

AAC calculates the frequencies of all 20 amino acids in a protein sequence.

APAAC incorporate partial sequence-order effect and correlation function by using the hydrophobicity and hydrophilicity properties of the constitute amino acids in a protein. Denoting the original hydrophobicity values of the 20 amino acids as $H_{1}^{O}$(i) (i = 1, 2, 3, …, 20). Similarly, the original hydrophobicity values is denoted as $H_{2}^{O}$(j). They are transformed to the following quantities by a standard conversion:

$\left\{ \begin{aligned} H_{1}\left( i \right)=\frac{H_{1}^{O}\left( i \right)-\frac{1}{20}\sum_{i=1}^{20} H_{1}^{O}\left( i \right)}{\sqrt{\frac{\sum_{i=1}^{20} \left[ H_{1}^{O}\left( i \right)-\frac{1}{20}\sum_{i=1}^{20} H_{1}^{O}\left( i \right) \right]^{2}}{20}}}, i=1,2,3,\ldots,20 \\ H_{2}\left( i \right)=\frac{H_{2}^{O}(i)-\frac{1}{20}\sum_{i=1}^{20} H_{2}^{O}(i)}{\sqrt{\frac{\sum_{i=1}^{20} \left[ H_{2}^{O}(i)-\frac{1}{20}\sum_{i=1}^{20} H_{2}^{O}(i) \right]^{2}}{20}}}, i=1,2,3,\ldots,20 \end{aligned} \right.$ (1)

the hydrophobicity and hydrophilicity correlation functions are defined as:

$H_{i,j}^{1}$=$H_{1}\left( i \right)H_{1}\left( j \right)$ (2)

$H_{i,j}^{2}$=$H_{2}\left( i \right)H_{2}\left( j \right)$ (3)

Thus, sequence order factors can be defined as:

$$\tau_{1}=\frac{1}{N-1}\sum_{i=1}^{N-1} H_{i,i+1}^{1}$$

$$\tau_{2}=\frac{1}{N-1}\sum_{i=1}^{N-1} H_{i,i+1}^{2}$$

$$\tau_{3}=\frac{1}{N-2}\sum_{i=1}^{N-2} H_{i,i+2}^{1}$$

$$\tau_{4}=\frac{1}{N-2}\sum_{i=1}^{N-2} H_{i,i+2}^{2}$$

…

$$\tau_{2\lambda-1}=\frac{1}{N-\lambda}\sum_{i=1}^{N-\lambda} H_{i,i+\lambda}^{1}$$

$$\tau_{2\lambda}=\frac{1}{N-\lambda}\sum_{i=1}^{N-\lambda} H_{i,i+\lambda}^{2}$$

Then, APAAC is defined as:

$P_{c}=\frac{f_{c}}{\sum_{r=1}^{20} f_{r}+w\sum_{j=1}^{2\lambda} \tau_{j}}, (1\leq c\leq20)$ (4)

$P_{c}=\frac{w\tau_{u}}{\sum_{r=1}^{20} f_{r}+w\sum_{j=1}^{2\lambda} \tau_{j}}, (21\leq u\leq20+2\lambda)$ (5)

where w is the weighting factor, we set w = 0.5.

DPC computes the frequencies of all dipeptides, which is defined as:

$D\left( r,s \right)=\frac{N_{rs}}{N-1}, r,s\in\{A, C, D\ldots Y\}$ (6)

where $N_{rs}$ is the number of dipeptides composed by r and s, which gives a 400-D vector.

The composition (C) feature characterizes the amino acid distribution patterns or physicochemical property in a protein. Twenty amino acids are categorized into three groups according to their physicochemical property (Additional file 5: Table S5). Taking the charge attribute for example, twenty amino acids are categorized into positive group (KR), neutral group (ANCQGHILMFPSTWYV) and negative group (DE). The three features of the composition descriptor represent the percentage of each group of residues in the protein sequence and is calculated as follows:

$\mathrm{CTDC}\left( r \right)=\frac{N(r)}{N}, r\in\{postive, neutral, negative\}$ (7)

where $N(r)$ is the number of amino acids of type r in a given sequence and N is the protein length.

The first 20 features (Equation 8) of the QSOrder represents the amino acid frequency, and the remaining features characterize the sequence order based on the Schneider-Wrede physicochemical distance matrix and the Grantham chemical distance matrix (Equation 9). It is defined as:

$X_{r}=\frac{f_{r}}{\underset{r=1}{\overset{20}{\sum}}f_{r}+w\underset{d=1}{\overset{nlag}{\sum}}\tau_{d}}, r=1, 2, 3, \ldots, 20$ (8)

$X_{d}=\frac{w\tau_{d-20}}{\underset{r=1}{\overset{20}{\sum}}f_{r}+w\underset{d=1}{\overset{nlag}{\sum}}\tau_{d}}, d=21,2 2, 23, \ldots,nlag$ (9)

$\tau_{d}=\sum_{i=1}^{N-d} {(d_{i,i+d})}^{2}, d=1, 2,3, \ldots, nlag$ (10)

where $f_{r}$ is the normalized occurrence of amino acid type r and weighting factor w = 0.1; $d_{i,i+d}$ is the distance between the two amino acids at position i and i + d in protein sequence; *nlag* is the maximum value of the lag, N is the protein length. Accordingly, the descriptor dimension will be 40+2×nlag.

Nineteen physicochemical features calculated by Biopython (15 features), TMHMM (3 features) and USEARCH (1 feature) were also used for sequence formulation, more detailes can be seen from Additional file 5: Table S6.
