# Supplementary material for: Prediction of protein solubility based on sequence physicochemical patterns and distributed representation information with DeepSoluE
Source: BMC Biol. 2023 Jan 24;21:12. doi: 10.1186/s12915-023-01510-8 (PMC9875434; doi:10.1186/s12915-023-01510-8)
Supplement: Supplementary file 5 — Additional file 5: Table S5. Thirteen types of physicochemical properties that used for computing the features of CTDC. Table S6. 15 physicochemical features calculated by Biopython and three features from TMHMM. Table S7. Hyperparameters for LSTM model. [file 12915_2023_1510_MOESM5_ESM.docx]

**Table S5** Thirteen types of physicochemical properties that used for computing the features of CTDC.

| **physicochemical properties** | **categorized groups** | | |
| --- | --- | --- | --- |
| Hydrophobicity_PRAM900101 | Polar: RKEDQN | Neutral: GASTPHY | Hydrophobicity: CLVIMFW |
| Hydrophobicity_ARGP820101 | Polar: QSTNGDE | Neutral: RAHCKMV | Hydrophobicity: LYPFIW |
| Hydrophobicity_ZIMJ680101 | Polar: QNGSWTDERA | Neutral: HMCKV | Hydrophobicity: LPFYI |
| Hydrophobicity_PONP930101 | Polar:KPDESNQT | Neutral: GRHA | Hydrophobicity:YMFWLCVI |
| Hydrophobicity_CASG920101 | Polar:KDEQPSRNTG | Neutral: AHYMLV | Hydrophobicity: FIWC |
| Hydrophobicity_ENGD860101 | Polar:RDKENQHYP | Neutral :SGTAW | Hydrophobicity: CVLIMF |
| Hydrophobicity_FASG890101 | Polar: KERSQD | Neutral: NTPG | Hydrophobicity:AYHWVMFLIC |
| Normalized van der Waals volume | Volume range: 0-2.78  GASTPD | Volume range: 2.95-94.0  NVEQIL | Volume range: 4.03-8.08  MHKFRYW |
| Polarity | Polarity value:4.9-6.2  LIFWCMVY | Polarity value: 8.0-9.2  PATGS | Polarity value: 10.4-13.0  HQRKNED |
| Polarizability | Polarizability value: 0-1.08  GASDT | Polarizability value:0.128-120.186  GPNVEQIL | Polarizability value: 0.219-0.409  KMHFRYW |
| Charge | Positive: KR | Neutral:ANCQGHILMFPSTWYV | Negative: DE |
| Secondary structure | Helix:EALMQKRH | Strand: VIYCWFT | Coil: GNPSD |
| Solvent accessibility | Buried:ALFCGIVW | Exposed: PKQEND | Intermediate: MPSTHY |

**Table S6** 15 physicochemical features calculated by Biopython and three features from TMHMM

| **Feature** | **Description** |
| --- | --- |
| fracnumcharge | Fraction of charged amino acids (R, K, D, E). |
| kr_ratio | Ratio of K and R content. |
| aa_helix | Fraction of helix amino acids (V, I, Y, F, W, L). |
| aa_sheet | Fraction of sheet amino acids (E, M, A, L). |
| aa_turn | Fraction of turn amino acids (N, P, G, S). |
| molecular_weight | Molecular weight. |
| avg_molecular_weight | Molecular weight normalized by the sequence length. |
| aromaticity | Fraction of aromatic amino acids (Y, W, F) |
| flexibility | Flexibility according to (Vihinen et al., 1994) |
| gravy | Grand average of hydropathy according to (Kyte and Doolittle, 1982) |
| isoelectric_point | Isoelectric point using methods of Bjellqvist |
| instability_index | Instability index according to (Guruprasad et al., 1990) |
| AAS43 | Amino acid profile calculated by scale (Cowan and Whittaker, 1990) |
| AAS38 | Amino acid profile calculated by scale (Janin, 1979) |
| AAS14 | Amino acid profile calculated by scale (Cowan and Whittaker, 1990) |
| AAs_TMHs | The expected number of amino acids intransmembrane helices |
| 60_AAs_TMHs | The expected number of amino acids in transmembrane helices in the first 60 amino acids of the protein |
| N_in_cytop | The total probability that the N-term is on the cytoplasmic side of the membrane |

**Table S7** Hyperparameters for LSTM model

| **Hyperparameters** | **Search range** |
| --- | --- |
| LSTM unite number for Layer | min_value=128, max_value=512, step=64 |
| Unite number for fully connected layer1 | min_value=128, max_value=256, step=32 |
| Unite number for fully connected layer2 | min_value=32, max_value=128, step=32 |
| Learning rate | [0.01,0.005,0.001,0.0005,0.0001,0.00005,0.00001,0.000005, 0.000001,0.0000005, 0.0000001] |
